# Hepatoprotective Effects of *Panus giganteus* (Berk.) Corner against Thioacetamide- (TAA-) Induced Liver Injury in Rats

**DOI:** 10.1155/2012/170303

**Published:** 2012-05-09

**Authors:** Wei-Lun Wong, Mahmood Ameen Abdulla, Kek-Heng Chua, Umah Rani Kuppusamy, Yee-Shin Tan, Vikineswary Sabaratnam

**Affiliations:** ^1^Mushroom Research Centre, Faculty of Science, University of Malaya, 50603 Kuala Lumpur, Malaysia; ^2^Institute of Biological Sciences, Faculty of Science, University of Malaya, 50603 Kuala Lumpur, Malaysia; ^3^Department of Molecular Medicine, Faculty of Medicine, University of Malaya, 50603 Kuala Lumpur, Malaysia

## Abstract

*Panus giganteus*, a culinary and medicinal mushroom consumed by selected indigenous communities in Malaysia, is currently being considered for large scale cultivation. This study was undertaken to investigate the hepatoprotective effects of *P. giganteus* against thioacetamide- (TAA-) induced liver injury in *Sprague-Dawley* rats. The rats were injected intraperitoneally with TAA thrice weekly and were orally administered freeze-dried fruiting bodies of *P. giganteus* (0.5 or 1 g/kg) daily for two months, while control rats were given vehicle or *P. giganteus* only. After 60 days, rats administered with *P. giganteus* showed lower liver body weight ratio, restored levels of serum liver biomarkers and oxidative stress parameters comparable to treatment with the standard drug silymarin. Gross necropsy and histopathological examination further confirmed the hepatoprotective effects of *P. giganteus*. This is the first report on hepatoprotective effects of *P. giganteus*. The present study showed that *P. giganteus* was able to prevent or reduce the severity of TAA-induced liver injury.

## 1. Introduction

The liver plays an important role in the detoxification of foreign substances, in the secretion of bile for digestion, and in the metabolic functions of various nutrients including carbohydrates, proteins, and fats [1]. Hence, chronic liver injury has serious medical consequences. A common chronic disease known as liver fibrosis may lead to end-stage liver cirrhosis and liver cancer [2]. Excessive consumptions of alcohol and viral infections are the most common risk factors for liver diseases in developed countries, while environmental pollution, hepatic viruses, parasitic infections, and chemotherapeutics are the main factors known to cause hepatic damage in developing countries [3].

In spite of medical advances, conventional medicinal approaches have undesirable adverse effects, lack efficiency, and are costly, especially for patients in developing countries [4]. Elimination of risk factors and alleviation of liver fibrosis are the most common approaches to prevent liver deterioration [5]. Therefore, there is an urgent need for safe alternative therapeutics to treat liver pathology. Many natural products are being targeted for liver disease prevention and/or treatment [1]. In recent years, mushrooms have been investigated for their potential for treating liver diseases [2, 6].

A mushroom is “a macrofungus with a distinctive fruiting body, which can be either hypogeous or epigeous, large enough to be seen with the naked eye and to be picked by hand" [7]. Mushrooms have been traditionally valued for their nutritional and pharmaceutical properties [8]. Mushrooms have been a major component of Chinese folk medicine since ancient times and it is only quite recently that scientists have begun investigating their bioactive compounds and health modulating mechanisms [9]. In Chinese traditional medicine, mushroom extracts are mixed with herbs in different combinations to treat various medical disorders [10].

Mushrooms have a huge potential in drug and nutraceutical development. They possess a wide range of pharmacological activities and thus can be considered a functional food. A large number of compounds with antimicrobial, antiviral, antioxidant, antitumor, antiallergic, anti-inflammatory, antiatherogenic, immunomodulating, hypoglycemic, hepatoprotective, and central activities has been characterized and isolated from mushrooms [9]. The dried fruiting bodies of mushrooms and extracts of mycelia grown in solid substrate and liquid fermentations are marketed as supplements in the form of powders, capsules, or tablets [11].

*Panus giganteus* (Berk.) Corner was introduced by mushroom growers and the Department of Agriculture, Malaysia, in 2003. It was previously known as “cendawan perut lembu” (cow's stomach mushroom) and has been renamed as lowland shiitake or “cendawan seri pagi” (morning glory mushroom). *Panus giganteus* is a known edible mushroom and widely consumed by the indigenous communities in Malaysia. Early studies have shown that *P. giganteus* can be easily cultivated in Malaysia. Large-scale cultivation of this mushroom looks promising with a good market price in Malaysia due to its delicate flavour. The cultivation of *P. giganteus* though relatively new in Malaysia is popular in China [12].

There is, however, a paucity of scientific data pertaining to its medicinal and nutritional benefits when compared to other edible mushrooms such as *Ganoderma lucidum* [13] and *Pleurotus sajor-caju *[14]. The aim of the present study was to evaluate the *in vivo* hepatoprotective effects of *P. giganteus* fruiting bodies against TAA-induced liver injury.

## 2. Materials and Methods

### 2.1. Mushroom Samples and Chemicals

*Panus giganteus* fruiting bodies collected from NAS Agrofarm Sdn. Bhd. were freeze dried (Christ freeze dryer Alpha 1-4 LD plus) and ground to a powder in a Waring commercial blender. The powder was then mixed with distilled water and administered orally to the experimental rats. In the present study, freeze-dried fruiting bodies were administered to the experimental rats to simulate human consumption of the *P. giganteus* fruiting bodies.

Silymarin was purchased from International Laboratory USA. According to Wills and Asha, silymarin as a standard drug has demonstrated excellent liver protection activity at a dose of 50 mg/kg [15]. Thus, a dose of 50 mg/kg was chosen in this experiment. Thioacetamide (TAA) and other chemicals were of analytical grade and purchased from Sigma-Aldrich or Fisher Scientific (M) Sdn. Bhd. Thioacetamide was dissolved in sterile distilled water at a concentration of 200 mg/kg body weight and injected intraperitoneally to the experimental rats [3]. The 8-hydroxy-2-deoxy Guanosine EIA detection kit was a product from Cayman Chemical (589320).

### 2.2. Experimental Rats

The experimental protocol was approved by the Animal Ethics Committee (ethic number: PM/28/08/2009/MAA (R)). All experimental rats were handled appropriately in accordance with the criteria prepared by the National Academy of Sciences Malaysia as outlined in the “Guide for the care and use of laboratory animals”. The *Sprague Dawley* rats of both sexes (200–250 g) were purchased from the Animal House Unit, Faculty of Medicine, University of Malaya, Malaysia. They were acclimated for three days prior to the experiment and were housed in specially prepared cages at 25 ± 3°C, 12 hours light-dark cycle with relative humidity of 50–60%. All the rats had free access to the standard diet and water *ad libitum*.

### 2.3. Acute Toxicity Study

Male and female *Sprague Dawley* rats were each divided into three different groups (*n* = 6) and assigned either as vehicle {sterile distilled water, 5 mL/kg, oral feeding (po)}, low-dose *P. giganteus* (2 g/kg, po), and high-dose *P. giganteus* (5 g/kg, po). The rats were not fed overnight prior to the treatments. After treatments, the rats were observed for toxicity symptoms and behavioural changes for a period of 48 hours. The observations continued up to day 14. Then, the rats were sacrificed after fasting overnight on the 15th day. Livers and kidneys were excised for gross necropsy and histopathological examination.

### 2.4. Hepatoprotective Effects of Freeze-Dried Fruiting Bodies Against TAA-Induced Liver Injury

Male and female* Sprague Dawley* rats were divided into six groups (*n* = 6) and subjected to various treatments for two months as depicted in Table 1.

The animals were weighed once a week and were observed for behavioural changes. At the end of the two-month treatment period, all rats were sacrificed under diethyl ether anesthesia after fasting overnight. Blood samples were collected and serum was isolated for biochemical assays. The livers were excised, rinsed in saline, blotted with filter paper, and weighed. Gross necropsy was performed to evaluate any abnormalities of the livers. Subsequently, the livers were processed for histopathological examination [3].

### 2.5. Effects of Different Treatments on Biochemical Parameters Related to Hepatoprotection

The blood samples were centrifuged at 3500 rpm (1534 ×g) for 10 minutes (Jouan C312 centrifuge). The resulting serum was then collected and sent to the Clinical Diagnostic Laboratory, University of Malaya Medical Centre to determine the liver biomarkers such as alkaline phosphatase (ALP), alanine aminotransferase (ALT), aspartate aminotransferase (AST), gamma-glutamyl transferase (GGT), bilirubin, total protein (TP), and albumin by using standard spectrophotometric measurements. Further, the determination of serum malondialdehyde (MDA) content was performed by thiobarbituric acid-reacting substances (TBARS) method as described by Daker et al. with minor modifications [16]. The results were calculated as 1,1,3,3-tetraethoxypropane (TEP) equivalents based on the TEP standard calibration. Oxidative damage of DNA was determined by measuring the levels of free 8-OH-dG in urine according to the protocol of the manufacturer (Cayman Chemical-589320). Urine was collected 24 hours before the rats were sacrificed and kept in −80°C freezer.

### 2.6. Histopathological Examination

Livers and kidneys from the experimental rats were sliced and fixed immediately after collection in 10% (v/v) formalin for at least 24 hours. The organs were then processed in an automated tissue-processing machine, embedded in paraffin, and cut into 5 *μ*m sections. Subsequently, the sections were stained with hematoxylin-eosin and observed under a microscope to evaluate histopathological changes.

### 2.7. Statistical Analysis

All results were expressed as mean ± S.E.M. (*n* = 6). The data was analysed by one-way analysis of variance (ANOVA) followed by Tukey's multiple comparison test. The level of significance was set at *P* < 0.05.

## 3. Results

### 3.1. Acute Toxicity Study

There was no morbidity and mortality observed throughout the study. *Panus giganteus* was not toxic to the experimental rats up to the high dose of 5 g/kg.

### 3.2. Effects of Different Treatments on Body and Liver Weights of Experimental Rats

The body and liver weights of the rats after two months of different treatments are shown in Table 2. Overall, there were no significant differences in body weight and liver weight between the rats in the different experimental groups. However, the rats treated with TAA exhibited significantly (*P* < 0.05) higher liver body weight ratios when compared to rats in control groups. The highest liver body weight ratio observed in TAA control rats was 73.71% higher than the ratio in the control rats (distilled water). Administration of *P. giganteus* (0.5 g/kg and 1 g/kg) lowered the liver body weight ratio and this was comparable to the effects observed in silymarin-administered rats (Table 2).

### 3.3. Effects of Different Treatments on Biochemical Parameters Related to Hepatoprotection

The changes in serum liver biomarkers are shown in Table 3. Rats in both control groups had similar biochemical indices. Particularly, TAA control rats exhibited the highest levels of ALP, ALT, AST, GGT, and bilirubin but lowest total protein and albumin content when compared to rats in other experimental groups. The serum ALP, ALT, AST, GGT, and bilirubin were 210.91%, 40.49%, 21.14%, 153.40%, and 198.75% higher when compared to serum levels in rats in the control group (distilled water). Total protein and albumin content dropped by 7.93% and 17.26%, respectively, when compared to rats in the control group (distilled water).

When a low dose of *P. giganteus* (0.5 g/kg) was administered, the levels of ALP, ALT, GGT, and bilirubin were significantly (*P* < 0.05) reduced while total protein and albumin content were significantly (*P* < 0.05) elevated. There were no significant differences in all the serum liver biomarkers of rats in the low-dose (0.5 g/kg) or high-dose (1 g/kg) treatment groups (Table 3). The rats administered with *P. giganteus* had significantly (*P* < 0.05) lower levels of ALP but comparable levels of ALT, AST, GGT, bilirubin, total protein, and albumin when compared to the rats administered with silymarin.

**Figure 1:**
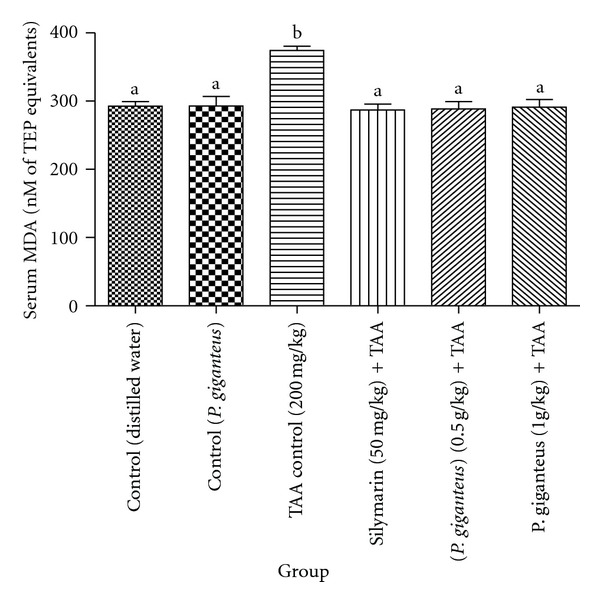
Comparison of serum MDA (nM of TEP equivalents) levels of experimental rats in different groups after two-month treatment. All values are expressed as mean ± S.E.M.; *n* = 6. Means with different letters (a-b) were significantly different at the level *P* < 0.05. MDA: malondialdehyde.

**Figure 2:**
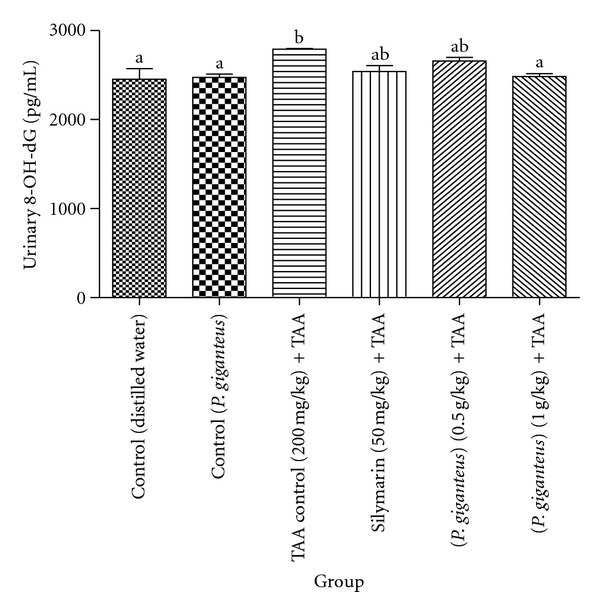
Comparison of urinary 8-OH-dG (pg/mL) levels of experimental rats in the different groups after two-month treatment. All values are expressed as mean ± S.E.M.; *n* = 6. Means with different letters (a-b) were significantly different at the level *P* < 0.05. 8-OH-dG: 8-hydroxydeoxyguanosine.

The levels of oxidative stress parameters (serum MDA and urinary 8-OH-dG) of the experimental rats are given in Figures 1 and 2. In general, rats in both control groups displayed similar levels of serum MDA and urinary 8-OH-dG. Notably, TAA control rats had significantly (*P* < 0.05) higher levels of MDA when compared to rats in other experimental groups and significantly (*P* < 0.05) higher urinary 8-OH-dG content when compared to rats in control groups and rats administered with a high dose of *P. giganteus*. *Panus giganteus* treatments reduced the serum MDA and urinary 8-OH-dG content. In addition, there were no significant differences in oxidative stress biomarkers between the rats administered with *P. giganteus* and those given silymarin.

### 3.4. Gross Necropsy and Histopathological Examination

**Figure 3:**
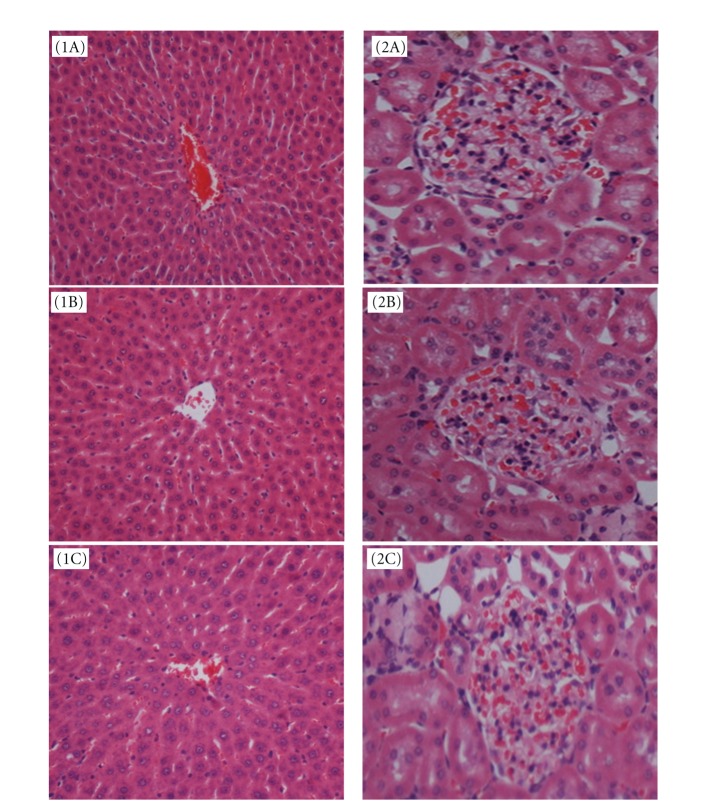
The photomicrography of liver and kidney sections of rats administered with *P. giganteus* at doses of 2 g/kg, 5 g/kg and distilled water. (1A and 2A) liver and kidney sections of control rat; (1B and 2B) liver and kidney sections of rat administered with low dose of *P. giganteus* (2 g/kg); (1C and 2C) liver and kidney sections of rat administered with high dose of *P. giganteus* (5 g/kg). (H&E stain, original magnification: 20x).

In the acute toxicity assay, gross necropsy and histopathological examination were performed on the livers and kidneys of the rats. There were no abnormalities or irregularities in the organs of all three experimental groups. The histopathological examination did not show any significant differences in cellular structures of livers and kidneys between the rats in the *P. giganteus* administered groups and those in the control group.  Figure 3 illustrates the histological sections of livers (1A, 1B, and 1C) and kidneys (2A, 2B, and 2C) in the acute toxicity study. Liver sections of rats administered with *P. giganteus* had a regular hepatic architecture. Distinct hepatic cells and well-preserved cytoplasm were observed. The kidney tissues retained the tubular structure and the cellular outlines were similar to rats in the control group.

**Figure 4:**
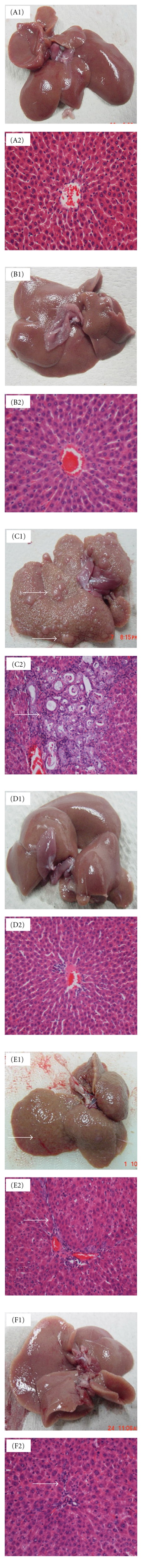
The gross liver morphology (A1–F1) and photomicrography of liver sections (A2-F2) of rats treated with TAA and administered with *P. giganteus* at doses of 0.5 g/kg, 1 g/kg, and distilled water. The rats in TAA, silymarin, and *P. giganteus* treatment groups were injected with TAA via the intraperitoneal-route thrice a week. (A1 and A2) control rat (distilled water); (B1 and B2) control rat (*P. giganteus*); (C1 and C2) TAA control rat: gross image shows many micro- and macronodules in the liver (arrow), while light micrograph shows thick fibrotic septae with proliferation of bile duct (arrow); (D1 and D2) Rat administered with silymarin. (E1 and E2) rat administered with low dose of *P. giganteus* (0.5 g/kg): micronodules were noted in the gross image (arrow), light micrograph shows narrow fibrotic septae (arrow); (F1 and F2) rat administered with high dose of *P. giganteus *(1 g/kg): very minor fibrotic septae was observed in the light micrograph (arrow). (Figure A2–F2: H&E stain, original magnification: 20x).

In the hepatoprotection experiment, gross necropsy and histopathological examination of liver tissues were correlated to the serum biochemical indices. Gross images of the livers are presented in Figure 4 (Al–F1) while Figure 4 (A2–F2) displays the histological sections of the livers. The livers of rats in both control groups had smooth surfaces without any irregularities (Figure 4: A1 and B1). Histological observations of the liver sections showed regular cellular architecture with distinct hepatic cells, sinusoidal spaces, and a central vein. The hepatic cells displayed prominent nuclei and uniform cytoplasm (Figure 4: A2 and B2).

In contrast, the livers of the rats in the TAA control group were enlarged with obvious inferior margins and contained many micro and macronodules (Figure 4: C1). The liver sections of the TAA control rats revealed extensive damage, characterized by severe necrosis, fatty degeneration, sinusoidal dilatation and congestion, centrilobular necrosis, proliferation of bile duct, presence of collagen bundles surrounding the lobules, which lead to thick fibrotic septae that disrupts the cellular architecture (Figure 4: C2). However, liver recovery was observed in rats administered with silymarin with liver condition and hepatic architecture similar to control groups (Figure 4: D1 and D2).

The liver enlargement and nodules were reduced in rats which were administered with low dose of *P. giganteus* (0.5 g/kg, Figure 4: E1). The histology of the liver sections in rats administered with low dose of *P. giganteus* showed significant improvement with less damage of liver tissue indicated by reduced level of necrosis, narrow fibrotic septae, remarkable increase in bile ductules, fat storing cells, and Kupffer cells (Figure 4: E2). Excellent liver recovery was indicated in rats administered with high doses of *P. giganteus* (1 g/kg) with liver morphology comparable to the control rats (Figure 4: F1). There was minimal disruption of the hepatic cellular structure; very minor fibrotic septae and a low degree of lymphocyte infiltration (Figure 4: F2).

## 4. Discussion

*Panus giganteus* did not have any adverse effects on experimental rats up to the high dose of 5 g/kg (equivalent to 28.57 g of fresh mushrooms) tested. There were neither mortality nor toxicity symptoms observed throughout the experiment. Gross necropsy and histopathological examination of the livers and kidneys further confirmed the nontoxicity of *P. giganteus*.

In the hepatoprotection experiment, although there were no significant changes in body and liver weights in the different experimental groups, higher liver body weight ratios had been observed in TAA-treated rats compared to rats in control groups. Measurement of liver body weight ratio is a more accurate approach to determine the changes in liver size compared to the measurement of liver weight alone as the liver weight largely depends on the size of the rat. The enlargement of livers in TAA treated rats signified hepatic lesions and liver injury associated with the toxicological effects of TAA. However, the liver enlargement was significantly (*P* < 0.05) reduced in rats administered with *P. giganteus* and this was comparable to the effects of silymarin.

Serum liver biomarkers (ALP, ALT, AST, GGT, bilirubin, total protein, and albumin) are important criteria for the evaluation of liver toxicity. The amounts of enzymes that leak into the blood stream indicate the severity of hepatic damage [17]. Additionally, the decreased level of albumin or hypoalbuminemia and total protein in TAA control rats could be due to malnutrition related to liver cirrhosis. In the present study, the rats intoxicated with thioacetamide (TAA) experienced hepatic injury evidenced by significant changes (*P* < 0.05) in serum liver biomarkers when compared to normal control rats. However, *P. giganteus* exhibited hepatoprotective effects to restore the altered serum liver parameters comparable to the effects of silymarin. This was further confirmed by gross necropsy and histopathological examination.

Generations of reactive oxygen species (ROS), mitochondrial dysfunction, and antioxidant insufficiency have been reported to advance the development of liver cirrhosis [18]. In order to evaluate the effects of TAA on oxidative stress in rats, we examined the oxidative stress parameters such as serum MDA and urinary 8-OH-dG content which reflect oxidative damage to lipids and DNA, respectively. Malondialdehyde (MDA) has been quantified since the 1960s and is still widely used as a biomarker to detect lipid peroxidation due to the low cost and simplicity of the application [19]. Numerous reports have revealed that the measurement of TBARS is useful to study the pathological states in tissues of animal origin [6, 20]. Earlier studies suggested that hepatotoxins including TAA, induced liver damage by forming free radicals, which then react with cellular lipids to promote lipid peroxidation [21]. The higher MDA level in TAA control rats observed in the present study also supports this suggestion.

The free 8-OH-dG assay was used for the evaluation of DNA damage caused by oxidative stress. The free radicals may damage nucleic acids, cellular lipids, and proteins at high concentrations [22]. Although oxidative damage of DNA results in a large number of different biochemical effects, the main focus has been on nucleobase alterations especially the lesion of 8-OH-dG as it is produced *in vivo* and can be estimated in cells after the DNA has been attacked by hydroxyl radicals [23]. Particularly, the urinary 8-OH-dG content has been most commonly used due to the simplicity and noninvasiveness of the method [24]. Kasai and Nishimura were the first to report the use of 8-OH-dG to analyse DNA damage caused by hydroxylation at the C8 position of the nucleoside guanosine [25]. Since then, numerous studies have been carried out and improvements have been made to the methods for the quantitation of 8-OH-dG in urine and plasma [26].

Our results indicate that TAA control rats experienced a higher degree of oxidative DNA damage than rats in the other experimental groups. The lower levels of serum MDA and urinary 8-OH-dG in rats administered with *P. giganteus* suggest that *P. giganteus* may possess bioactive compounds that could prevent the oxidative stress induced by TAA and thus alleviate the degree of liver injury. Furthermore, as there were no remarkable differences between the high dose (1 g/kg, equivalent to 5.72 g of fresh mushrooms) and the low dose (0.5 g/kg, equivalent to 2.86 g of fresh mushrooms) treatments with regard to their serum biochemical indices and histopathological evidences, it is evident that the low dose of *P. giganteus* had protective effects against TAA-induced liver deterioration. *Panus giganteus* may be incorporated into the diet of patients with liver disease as the present study showed that it may reduce the severity of liver lesions, while exhibiting no toxic effects to rats in animal experiments.

Silymarin was used as a positive control because silymarin is a standard drug exhibiting excellent liver protection activity [3, 15]. Hepatotoxic agents such as acetaminophen [27], carbon tetrachloride [28], galactosamine [29], and thioacetamide (TAA) [3] have been used to induce experimental liver damage in both *in vivo* and *in vitro* study models. These hepatotoxic agents induce liver damage by reacting with basic cellular components [30]. Thioacetamide (TAA) as a thiono-sulfur-containing compound has been widely used as a fungicide, organic solvent, accelerator in the rubber vulcanization, and as motor oil stabilizer [31]. Fitzhugh and Nelson were the first to report TAA as a hepatotoxic agent [32]. Thioacetamide binds covalently to the cellular components and subsequently induces oxidative stress [33, 34]. Biochemically, it is metabolized by the microsomal FAD monoxygenase (FADM) system to form reactive metabolites such as thioacetamide sulfoxide and thioacetamide-S,S-dioxide which then contribute to the toxicity effects of TAA [35]. Long-term exposure to TAA may lead to hyperplastic liver nodules, liver cell adenomas, and hepatocarcinomas [36].

According to Pérez et al. [37], metabolic and histological changes in cirrhotic models induced by TAA closely resemble those in human liver cirrhosis. Thioacetamide- (TAA-) induced liver cirrhosis in rats served as a good model as it reflects major human physiological changes. Moreover, the use of TAA is more appropriate in such experiments due to its high specificity to the liver, regiospecificity to the perivenous area, and having a large window of time between its hepatocytes damaging effects and liver failure [38].

In the present study, the mushroom was administered at doses of 0.5 g/kg (low dose) and 1 g/kg (high dose) as previous related studies indicated that these doses would be effective in preventing liver injury. Dai et al. showed that oral administration of *Antrodia camphorata* fruiting bodies in doses of 0.5 g/kg and 1 g/kg provided protection against ethanol-induced acute liver damage in *Sprague-Dawley* rats [39]. Lu et al. suggested the remarkable preventive effects of 0.5 g/kg and 1 g/kg *Antrodia camphorata *mycelia in submerged culture against ethanol-induced hepatic toxicity in *Sprague-Dawley* rats [40]. In addition, other mushroom species including *Ganoderma lucidum* [6] and *Pleurotus ostreatus* [20] exhibited hepatoprotective effects using rats as experimental model.

## 5. Conclusions

In conclusion, the acute toxicity study showed that *P. giganteus* was not toxic to the experimental rats up to an oral dose of 5 g/kg body weight. Furthermore, *P. giganteus* exhibited significant hepatoprotective effects against TAA-induced liver injury in rats even at a low dose of 0.5 g/kg and was comparable to the effects of silymarin, a standard drug used to treat liver diseases. The hepatoprotective effects of *P. giganteus* were associated with its ability to reduce oxidative stress. However, further investigation is required to identify the bioactive compounds responsible for the hepatoprotective effects and to formulate functional foods for the reduction of liver injury severity.

## Figures and Tables

**Table 1:** The various treatments to assess the hepatoprotective effects of *P. giganteus* during the two-month study.

No.	Group	Treatment
1	Control (distilled water)	Sterile dH_2_O (5 mL/kg, po) daily; sterile dH_2_O (5 mL/kg, i.p) thrice weekly
2	Control (*P. giganteus*)	*Panus giganteus *(0.5 g/kg, po) daily; sterile dH_2_O (5 mL/kg, i.p) thrice weekly
3	TAA control (200 mg/kg)	Sterile dH_2_O (5 mL/kg, po) daily; TAA (200 mg/kg, i.p) thrice weekly
4	Silymarin (50 mg/kg) + TAA	Silymarin (50 mg/kg, po) daily; TAA (200 mg/kg, i.p) thrice weekly
5	*Panus giganteus* (0.5 g/kg) + TAA	*Panus giganteus *(0.5 g/kg, po) daily; TAA (200 mg/kg, i.p) thrice weekly
6	*Panus giganteus* (1 g/kg) + TAA	*Panus giganteus *(1 g/kg, po) daily; TAA (200 mg/kg, i.p) thrice weekly

po: oral feeding; i.p: intraperitoneal injection.

**Table 2:** Effects of different treatments on body and liver weights of experimental rats.

Group	Body weight, BW (g)	Liver weight, LW (g)	LW/BW (%)
Control (distilled water)	384.50 ± 36.57^b^	9.69 ± 0.99^ab^	2.51 ± 0.04^a^
Control (*P. giganteus*)	370.33 ± 41.87^ab^	9.92 ± 1.10^ab^	2.70 ± 0.08^a^
TAA control (200 mg/kg)	296.17 ± 18.47^ab^	12.85 ± 0.87^b^	4.36 ± 0.20^c^
Silymarin (50 mg/kg) + TAA	265.00 ± 15.53^a^	8.55 ± 0.75^a^	3.21 ± 0.13^b^
*Panus giganteus *(0.5 g/kg) + TAA	283.33 ± 17.06^ab^	10.28 ± 0.54^ab^	3.64 ± 0.09^b^
*Panus giganteus *(1 g/kg) + TAA	306.33 ± 18.79^ab^	10.55 ± 0.63^ab^	3.45 ± 0.09^b^

Two-month treatment; food and water *ad libitum*. All values are expressed as mean ± S.E.M.; *n* = 6. Means with different letters (a–c) were significantly different at the level *P* < 0.05.

**Table 3:** Effects of different treatments on serum liver biomarkers of experimental rats.

Group	ALP (IU/L)	ALT (IU/L)	AST (IU/L)	GGT (IU/L)	Bilirubin (*μ*mol/L)	Total protein (g/L)	Albumin (g/L)
Control (distilled water)	70.17 ± 5.62^a^	46.50 ± 3.04^a^	164.00 ± 14.68^a^	5.00 ± 0.26^a^	2.40 ± 0.20^a^	69.33 ± 0.92^cd^	13.50 ± 0.34^b^
Control (*P. giganteus*)	79.17 ± 6.87^a^	46.00 ± 1.59^a^	151.00 ± 3.42^a^	5.20 ± 0.40^a^	2.60 ± 0.20^a^	70.83 ± 0.79^d^	13.83 ± 0.17^b^
TAA control (200 mg/kg)	218.17 ± 5.47^c^	65.33 ± 0.67^c^	198.67 ± 0.21^b^	12.67 ± 1.87^c^	7.17 ± 0.83^c^	63.83 ± 0.48^a^	11.17 ± 0.40^a^
Silymarin (50 mg/kg) + TAA	207.00 ± 11.93^c^	56.33 ± 2.19^b^	182.00 ± 5.82^ab^	10.00 ± 0.52^bc^	5.20 ± 0.31^b^	65.50 ± 0.56^ab^	12.83 ± 0.48^ab^
*Panus giganteus *(0.5 g/kg) + TAA	165.67 ± 5.67^b^	55.17 ± 2.34^b^	174.00 ± 8.80^ab^	7.17 ± 0.91^ab^	5.00 ± 0.26^b^	67.00 ± 0.26^bc^	14.00 ± 0.68^b^
*Panus giganteus *(1 g/kg) + TAA	166.67 ± 9.19^b^	53.33 ± 0.80^ab^	164.00 ± 4.37^a^	6.83 ± 0.60^ab^	4.80 ± 0.16^b^	67.17 ± 0.60^bc^	13.83 ± 0.40^b^

Two month treatment; food and water *ad libitum*. All values are expressed as mean ± S.E.M.; *n* = 6. Means with different letters (a–d) were significantly different at the level *P* < 0.05. ALP: alkaline phosphatase; ALT: alanine aminotransferase; AST: aspartate aminotransferase; GGT: gamma-glutamyl transferase.
